# Cesarean section indications and anthropometric parameters in Rwandan nulliparae: preliminary results from a longitudinal survey

**DOI:** 10.11604/pamj.2016.24.310.9603

**Published:** 2016-08-12

**Authors:** Jean-Baptiste Kakoma

**Affiliations:** 1 University of Lubumbashi Faculty of Medicine and School of Public Health, Lubumbashi, Democratic Republic of the Congo; 2 University of Rwanda, College of Medicine and Health Sciences, Schools of Medicine and Public Health, Kigali, Rwanda

**Keywords:** Cesarean section indications, anthropometric parameters, nulliparae, Rwanda

## Abstract

**Introduction:**

Maternal anthropometric parameters as risk factors for cesarean section have always been a matter of interest and concern for obstetricians. Some of these parameters have been shown to be predictors of dystocia. This study aims at showing the relationship between cesarean section indications and anthropometric parameters sizes in Rwandan nulliparae for the purpose of comparison and appropriate recommendations.

**Methods:**

A cross-sectional and analytical study was made on data collected from 32 operated patients among 152 nulliparae with singleton pregnancy at term and vertex presentation. Concerned anthropometric parameters were height, weight and six pelvic distances. Fisher exact and Student’s tests were used to compare observed proportions and mean values, respectively.

**Results:**

Findings were as follows: 1) the overall cesarean section rate was 21.05%; 2) acute fetal distress (31.3 %), generally contracted pelvis (28.1 %), and engagement failure (25%) were the most frequent indications of cesarean section; 3) all patients ≤ 145 cm tall were operated on for general pelvis contraction whose proportion was significantly higher in them than in the others (p < 0.01); 4) more than half of pelvis contraction cases were observed in patients weighing ≤ 50 kg, but the difference with other weight categories was not significant; 5) considered external pelvic diameters but the Biiliac Diameter displayed average measurements smaller in clinically contracted pelvis than in other CS indications.

**Conclusion:**

External pelvimetry associated with specific other anthropometric parameters could be helpful in the screening of generally contracted pelves, and consequently pregnancies at high risk of cephalopelvic disproportion in nulliparous women, particularly in developing countries with limited resources. Further investigations are requested to deal with this topic in depth.

## Introduction

Maternal anthropometric parameters (i.e. height, weight, body mass index and external pelvic diameters) as risk factors for cesarean section have been for a long time a matter of interest and concern for obstetricians [1–14]. Furthermore, some of these parameters have been shown to be predictors of dystocia due to cephalopelvic disproportion [3, 8, 13] or contracted pelvis [1], thus leading to lower segment cesarean section in nulliparous women. The absence of well-documented data in that field has justified a number of studies in Rwandan nulliparae from an extensive survey on pelvimetry and other anthropometric parameters. Research findings from one of these studies have highlighted some discrepancies about cesarean section rate distribution as per considered anthropometric parameters, namely height, weight and main external pelvic diameters, due to some supposed bias factors [15]. Cesarean section indications were among these suggested bias factors, hence this study, which is a further analysis of above-mentioned study data. It aims at showing the relationship between anthropometric sizes and cesarean section indications in Rwandan nulliparae. Its findings will be next compared to those from elsewhere, and Central African region especially. Ultimately, recommendations will be made towards a simple, efficient and predictor tool for unavoidable timely cesarean sections.

## Methods

A cross-sectional and analytical study was made on data collected from 32 operated parturients among 152 nulliparae who gave birth with singleton pregnancy at term and vertex presentation within the three first months of a prospective longitudinal survey in the Southern Province of Rwanda. This prospective survey consisted in a close follow-up of a cohort of Rwandan nulliparae (versus multiparous controls who never experienced cesarean section) from first antenatal care visit to delivery in order to match ways of delivery and sizes of concerned anthropometric parameters. Anthropometric measurements were collected at first antenatal care consultation (height and external pelvic diameters in cm) and at admission in the labor room (weight in kg). At childbirth, midwives and physicians monitored labor in a blind way and the following data were collected after delivery by the investigators: way of delivery (per vaginam or cesarean section), and cesarean section indication. Fisher exact test and Student’s t test were used to respectively compare observed proportions and mean values from a normally distributed population. The difference was considered significant for p < 0.05.

## Results

### Indications of cesarean sections

Indications of the 32 cesarean sections, which represented a prevalence of 21.05% for the study population, were as follows: acute fetal distress (AFD: 10 = 31.3%), generally contracted pelvis (GCP: 9 = 28.1%), engagement failure (EF: 8 = 25%), cephalopelvic disproportion (CPD: 3 = 9.4%), face presentation (FP: 1 = 3.1%) and eclampsia (Ec: 1 = 3.1%).

### Height and cesarean section indications

All patients ≤ 145 cm tall underwent cesarean section for general pelvis contraction. GCP proportion decreased when height increased and became null beyond 160 cm. The difference between GCP proportions observed in patients ≤ 145 cm tall and the others (100% versus 17.86%) was highly significant. In the same way, the difference between patients ≤ 145 cm tall and those > 160 cm tall (100% versus 0%) was significant. The average height was 149.78 (± 7.68) cm in patients presenting a GCP and 152.33 (± 3.06) cm in those with cephalopelvic disproportion; the difference was not significant (p = 0.7352). Five out of the eight cases of engagement failure presented a height not exceeding 160 cm (Table 1).

**Table 1:** Height and cesarean section indication in Rwandan nulliparae

HEIGHT (cm)	GCP	AFD	FP	Ec	EF	CPD	TOTAL
**≤145**	4	-	-	-	-	-	4
**146-150**	1	1	-	-	-	1	3
**151-155**	1	5	-	-	2	2	10
**156-160**	3	4	-	1	3	-	11
**>160**	-	-	1	-	3	-	4
**TOTAL**	9	10	1	1	8	3	32

Fisher exact test for GCP: ≤ 145 cm class versus all other classes: p = 0.003504** ≤ 145 cm class versus > 160 cm class: p = 0.028571*

### Weight and cesarean section indications

More than half of general pelvis contraction cases were observed in patients weighing ≤ 50 kg. GCP proportion decreased in upper weight categories. However, the difference in GCP proportion was not significant either between = 50 kg category and the others (57.14% versus 20%) or between = 50 kg and > 60 kg categories (57.14% versus 10%). The average weight in patients presenting GCP was 52.22 (± 6.04) kg. Even if patients weighing = 50 kg seemed to be prone to GCP, the difference with others was not significant (**Table 2**).

**Table 2:** Weight and cesarean section indication in Rwandan nulliparae

WEIGHT (Kg)	GCP	AFD	FP	Ec	EF	CPD	TOTAL
≤50	4	2	-	-	1	-	7
51-55	4	2	1	-	2	1	10
56-60	-	2	-	-	2	1	5
>60	1	4	-	1	3	1	10
TOTAL	9	10	1	1	8	3	32

Fisher exact test for GCP: ≤ 50 kg class versus all other classes: p = 0.076353 ≤ 50 kg class versus > 60 kg class: p = 0.100679

### Patients’ external pelvimetry and cesarean section indications

All considered external pelvic diameters (biiliac, antero-superior iliac interspinous, intertrochanter, Baudelocque’s, and intertuberous) and the base length of the Trillat’s triangle displayed average measurements smaller in clinically diagnosed pelvis contraction than in other CS indications. Apart from biiliac diameter, observed differences were all statistically significant (Table 3).

**Table 3:** External pelvimetry and cesarean section indication in Rwandan nulliparae

DIAMETERS (cm)	Contracted pelvis (N = 9)	Other indications (N = 23)	Mean difference	p
**BIILIAC**	22.83 ± 2.59	23.45 ± 1.34	0.62	0.375
**ANTERO– SUPERIOR ILIAC INTERSPINOUS**	20.87 ± 1.59	21.94 ± 1.15	1.07	0.043
**INTERTROCHANTER**	25.70 ± 2.56	27.72 ± 2.26	2.02	0.036
**BAUDELOCQUE**	16.20 ± 1.17	18.50 ± 1.31	2.30	0.000
**INTERTUBEROUS**	7.76 ± 0.43	9.26 ± 0.68	1.50	0.000
**TRILLAT’S BASE**	12.17 ± 2.26	13.98 ± 0.93	1.81	0.003

## Discussion

Cesarean section rate (21.05%) in our nulliparous study population was higher than the upper limit (15%) for cesarean section set by WHO [16] and by far lower than those registered for the two previous years in a urban national reference hospital (41%) [17] and a rural district hospital (33.7%) of Rwanda [18]. Parturients’ height ≤ 145 cm was significantly characterized by generally contracted pelvis leading to unavoidable cesarean section in pregnancies at term whereas heights exceeding 160 cm seemed to be free from GCP in our study population. Besides, all three CPD indications in the present study were related to heights between 146 and 155 cm inclusive. Height < 150 cm in nulliparae has been identified as a risk factor for cesarean section and cephalopelvic disproportion in urban and rural populations of different Western, Central and North–Eastern parts of the neighbouring Democratic Republic of the Congo [8, 9]. This same height cut-off size has also been observed elsewhere [11]. A cut-off size of < 140 cm has been reported from a well-documented survey in New Zealand [7]. A maternal height <160 cm has been nonetheless shown to be associated with an increased risk of CPD as compared to taller women in Zimbabwe [3]. Shorter maternal height has been identified as one of the risk factors for operative vaginal delivery in nulliparous women [3, 4, 7], and associated to increased risk of emergency cesarean section due to obstructed labour [7]. Cesarean section rates have been found to decrease with increasing maternal height [5], while cesarean section rate increased gradually with decreasing height [7]. However, an association of increased cesarean section risk with maternal age has also been reported in a tallest group of women [5].

Besides, the likelihood of having a normal delivery with measurements lower than 140 cm should not be excluded as evidenced by the case of some ethnic groups in the Democratic Republic of the Congo, former Belgian Congo and Ruanda – Urundi. Measurements for the Basua pygmies of the Ngayu region (North Eastern region of DR Congo) have shown in parturient women an average height and extremes of 138.4 cm and 118.4-151.5 cm respectively, with newborn at term and normal delivery [19]. The same observation was made in New Zealand [7]. Nulliparae weighing ≤ 50 kg at delivery seemed to be also correlated with generally contracted pelvis in our study population. However, delivery low maternal weight (i.e. < 100 pounds = 45.45 kg) was not correlated with cesarean section but active – phase arrest, preterm labor and delivery, and mediolateral episiotomy from a perinatal database study in a high-risk obstetrical and neonatal intensive care center [10]. An extensive literature, which cannot be cited on the whole, has been dedicated to maternal weight relationship with mode of delivery. Increase of cesarean section rates has been reported with higher prepregnancy weights [5] or prepregnancy maternal corpulence [12], an increasing body mass index and greater gestational weight gain [6], morbid obesity significantly requiring an emergency cesarean section [14]. It is worth pointing out that most of studies establish a significant relationship between increased body mass index and cesarean section, whereas leanest mothers have the best rate of vaginal delivery [12]. An average weight and extremes as low as 37.0 and 25-48 kg respectively were found to be compatible with vaginal delivery in female pygmies in DR Congo [19]. This could be explained by the fact that there could be a certain adaptability of fetal birthweights to maternal corpulence [12].

As for external pelvimetry in the current study, significantly lower measurements were displayed in generally contracted pelves that have been clinically diagnosed during labor by health professionals who were not involved in the survey. Apart from average values that did not match, results of this study were similar to findings concerning the relationship between some lower pelvic diameters (e.g. Baudelocque’s, interspinous, and intertrochanter) and cephalopelvic disproportion in Congolese nulliparae [8]. For many years, a reduced external conjugate (Baudelocque’s) diameter was used as an index of contracted pelvis [1]. However, if one refers to the case of female pygmies in DR Congo, a Baudelocque’s diameter of 15.6 cm (versus 20 cm in European women) did not prevent the fetal head to easily go through the mother’s pelvis whose sizes were 4-7 cm smaller than those of her European counterpart [20]. Two explanations were suggested: either internal sizes of pygmies’ pelves are not different from those of both Europeans’ and surrounding Bantu ethnic groups’ pelves or pygmies’ pelves joints greatly relax during delivery, given the very few cases of dystocia and cesarean section in this ethnical group [20]. The same findings were recorded from neighbour Wanande women whose pelves were platypelloid and generally contracted in comparison with the European anatomical configuration, but with comparatively smaller newborns [21]. And yet it is known that cephalopelvic disproportion results from mismatch between the size of the fetal head and the maternal pelvic size, hence engagement failure during labor for mechanical reasons [22]. As one risk factor alone is unlikely to affect delivery management regarding the relationship between anthropometric parameters and way of delivery [7], taking into consideration risk factors associations, as already initiated by some authors [8, 13], should be recommended to come up with a simple, efficient and predictor tool. This would allow performing well-timed unavoidable cesarean sections. Consequently, further investigations in different geographical environments of limited resources countries should be promoted, although it has been shown that clinical pelvimetry findings are not at all exploited in practice by general practitioners and obstetricians.

## Conclusion

Despite a number of controversial considerations, there could be room for external pelvimetry associated with specific other anthropometric parameters (i.e. height, weight, Body Mass Index, mid upper arm circumference…) to be helpful - at antenatal care consultations and delivery room admission in the screening of generally contracted pelves, and consequently pregnancies at high risk of cephalopelvic disproportion, particularly in developing countries with limited resources. The narrowness of the study population sample size is the major limitation factor for this study whose relevance needs to be confirmed through a more extended and in depth survey.

### What is known about this topic

- Significantly smaller height (< 150 cm) in cephalopelvic disproportion than in normal delivery and other complicated deliveries; maternal height < 140 cm compatible with normal delivery in some human groups; and maternal height <160 cm associated with an increased risk of CPD as compared to taller women;
- Relationship between prepregnancy maternal corpulence / increased body mass index / greater gestational weight gain and cesarean section;
- Significantly smaller pelvic diameters (but intertuberous diameter) in cephalopelvic disproportion than in normal delivery and other complicated deliveries.

### What this study adds

- First publishable study in Rwandan women anthropometry in relation to cesarean section indications;
- Height ≤ 145 cm as cut-off size for cesarean section in the population of Southern Province of Rwanda;
- Relationship between generally contracted pelvis indication, reduced external pelvic mensurations (including intertuberous diameter and base length of the Trillat’s triangle) and cesarean section.
